# Neuroblastoma in monozygotic twins – a case of probable twin-to-twin metastasis

**DOI:** 10.1054/bjoc.2001.1979

**Published:** 2001-08

**Authors:** J Anderson, H Kempski, L Hill, D Rampling, T Gordon, A Michalski

**Affiliations:** Units of Molecular Haematology and Cancer Biology; Department of Histopathology, Institute of Child Health and Great Ormond Street Hospital for Children NHS Trust, London; Department of Cytogenetics, St George's Hospital Medical School, London; Institute of Cancer Research, Sutton, Surrey, UK

**Keywords:** neuroblastoma, monozygotic twins

## Abstract

Concordance for neuroblastoma in monozygotic twins has been reported only rarely, and the cause of the shared pathology has not been established. We describe a case of infant monozygotic twins developing tumours that were morphologically, clinically and molecularly indistinguishable, but with a delay of 6 months between times of presentation. Both tumours were metastatic and had amplification of MYCN and deletion at 1p36. Twin 1, who developed neuroblastoma first, had constitutional karyotype abnormalities in at least 5% of peripheral blood mononuclear cells involving 1p and 3p, and a deletion of 1q44 in 21% of cells. Twin 2 had a normal constitutional karyotype and lacked rearrangement or deletion of these regions. We propose an acquired neuroblastoma predisposition specific for twin 1, and in utero metastatic spread of tumour cells to twin 2 via the shared placental circulation. © 2001 Cancer Research Campaign http://www.bjcancer.com


*British Journal of Cancer* (2001) **85**(4), 493–496
© 2001 Cancer Research Campaign
doi: 10.1054/ bjoc.2001.1979, available online at http://www.idealibrary.com on http://www.bjcancer.com

### Neuroblastoma in monozygotic twins – a case of probable twin-to-twin metastasis

**J Anderson[1]*, H Kempski[1]*, L Hill[3], D Rampling[2], T Gordon[4] and A Michalski[1]**

[1]Units of Molecular Haematology and Cancer Biology; [2]Department of Histopathology, Institute of Child Health and Great Ormond Street Hospital for Children NHS Trust, London; [3]Department of Cytogenetics, St George's Hospital Medical School, London and [4]Institute of Cancer Research, Sutton, Surrey, UK.

**Summary** Concordance for neuroblastoma in monozygotic twins has been reported only rarely, and the cause of the shared pathology has not been established. We describe a case of infant monozygotic twins developing tumours that were morphologically, clinically and molecularly indistinguishable, but with a delay of 6 months between times of presentation. Both tumours were metastatic and had amplification of MYCN and deletion at 1p36. Twin 1, who developed neuroblastoma first, had constitutional karyotype abnormalities in at least 5% of peripheral blood mononuclear cells involving 1p and 3p, and a deletion of 1q44 in 21% of cells. Twin 2 had a normal constitutional karyotype and lacked rearrangement or deletion of these regions. We propose an acquired neuroblastoma predisposition specific for twin 1, and in utero metastatic spread of tumour cells to twin 2 via the shared placental circulation. © 2001 Cancer Research Campaign http://www.bjcancer.com

**Keywords**: neuroblastoma; monozygotic twins

Neuroblastoma in monozygotic twins is rarely reported but of interest because of the insights it gives regarding tumorigenesis and/or metastasis. Previously, only 6 sets of monozygotic twins concordant for neuroblastoma have been described and none of concordant dizygotic twins (Boyd and Schofield, 1995; Kushner and Helson, 1985; Mancini et al, 1982). This relative absence, together with the paucity of constitutional karyotype abnormalities, suggests that there is not, in general, a strong constitutional genetic component for neuroblastoma (Buckley et al, 1996). Rare descriptions of constitutional rearrangements involving regions 1p36 and 17q11-ter have confirmed the importance of these regions in the aetiology of neuroblastoma (Biegel et al, 1993; Laureys et al, 1990; Laureys et al, 1995a; Mead and Cowell, 1995). The general lack of predisposing constitutional factors coupled with the relatively high incidence of blood-borne metastatic disease in congenital neuroblastoma, raises the intriguing possibility of twin-to-twin metastasis in concordant twins. This pattern of transplacental dissemination has been proven in cases of infant leukaemia, but never previously reported in a solid tumour. We report a further case of twins concordant for neuroblastoma and we propose transplacental metastatic spread as the most likely mechanism of shared pathology.

### MATERIALS AND METHODS

G banding of metaphases derived from peripheral blood samples were prepared using standard techniques. A total of 52 metaphase cells were examined from twin 1, and 49 from twin 2.

Slides for fluorescence in situ hybridization (FISH) were prepared from fixed cell samples and pre-treated using standard techniques. Whole chromosome paints (WCPs) for chromosomes 1 and 3 (Cambio, Cambridge, UK) and probes for MYCN, distal chromosome 17q, the telomeric region of 1q (VYSIS Inc, Illinois, USA), the sub-telomeric region of 1q (1q44-qter) (Appligene Oncor, Co. Durham, UK) and the p58 cosmid probe that maps to 1p36 (Appligene Oncor, Co. Durham, UK) were prepared and hybridized according to the manufacturers' instructions. For analysis by WCPs of chromosomes 1 and 3 rearrangements, 43 metaphase cells were examined from twin 1 and 47 from twin 2. For the locus-specific probes for chromosome 1, 87 metaphase cells were examined from twin 1, and 53 from twin 2.

### CASE HISTORY

The patients were baby girls born following a healthy pregnancy at 37 weeks' gestation to unrelated parents who had 3 older, healthy children. At delivery there was a single placenta. At 3 weeks of age, twin 1 presented with abdominal swelling, hepatomegaly, a left suprarenal mass, 10% infiltration of small round cells in the bone marrow and raised levels of urinary catecholamines and serum LDH and NSE. A biopsy of the adrenal tumour gave a histological diagnosis of neuroblastoma.

In view of the previous reports of concordance for neuroblastoma in monozygotic twins, it was decided that twin 2 would be screened by assessment of urinary catecholamines as well as by abdominal ultrasound scan every 3 months. At 6 months of age, a $3.5 \times 5.0$ cm right adrenal mass and 3 nodular hepatic lesions were found, although catecholamine levels were normal and there was no evidence of lesions at other metastatic sites. An open biopsy of the adrenal tumour gave a diagnosis of neuroblastoma, which was histologically indistinguishable from that of her sister.

Twin 1 presented with a rapidly growing tumour causing cardiorespiratory failure and requiring ventilatory and inotropic support until there was tumour shrinkage. Both twins had been

*Received 15 November 2000*
*Revised 01 May 2001*
*Accepted 16 May 2001*

*Correspondence to:* J Anderson

*These authors contributed equally to this project treated with initial intensive chemotherapy (courses of vincristine, cyclophosphamide, etoposide and carboplatin alternating with identical courses substituting cisplatin for carboplatin), surgery of the primary tumour, consolidation of remission with high-dose melphalan and continuation treatment with 13-cis retinoic acid. At the time of writing both twins remain well and disease free 18 and 12 months, respectively, following completion of treatment.

### RESULTS

Only a small needle tumour biopsy was available for twin 1. Interphase FISH on touch imprints from this tumour revealed multiple copies of the MYCN oncogene and deletion of chromosome region 1p36. Twin 2's tumour was investigated by both interphase and metaphase FISH. Interphase showed multiple copies of MYCN and deletion of 1p36. G banding of metaphases revealed an apparently normal karyotype, but addition of locus-specific flourescent probes demonstrated amplification of MYCN on double minutes, loss of chromosome region 1p36 (up to 8 copies of the chromosome 1 centromeric region but only ever 1 copy of 1p36), and gain of distal chromosome 17q (consistently 2 copies of 17 centromere but mostly 3 copies of both ERBB2 and MPO probes). Gain at 17q and 2p23-24 was confirmed for this tumour by comparative genomic hybridization analysis. Constitutional DNA from both twins was used in microsatellite analysis of highly polymorphic CA repeats and confirmed monozygosity.

Constitutional karyotyping of twins 1 and 2 was performed at 10 and 4 months, respectively, following high-dose melphalan treatment. There were abnormalities of chromosome 1 and/or chromosome 3 in 3 out of 52 (5.7%) of cells from twin 1 (Table 1). The complex internal rearrangement of chromosome 1, in one cell only, appeared to have resulted from the loss of the p arm segment from band 1p13.1→1p36, followed by duplication of the q arm segment from band q21→q44, followed by the pericentric inversion and insertion of the duplicated segment into the region of deletion at 1p13.1→1p36. A paracentric inversion of chromosome 3 between bands p23 and p26 was detected in the same cell containing the derivative chromosome 1 and in a further 2 metaphase cells, which appeared to have normal copies of chromosome 1. FISH analysis with WCPs revealed 2 out of 43 (4.6%) metaphase cells with abnormal copies of 1 chromosome 1. In both cells, the breakpoint on chromosome 1 appeared to be at 1p13.1, with the suggestion that a translocation partner was involved, possibly chromosome 17. However this could not be verified with the subsequent use of a WCP for 17. A subtelomeric probe for chromosome 1q disclosed deletions of this region in 24 out of 111 (21.6%) metaphase cells. Later investigation with a probe for the telomeric region of 1q demonstrated the presence of telomeric sequences in all cells examined. There was no indication of deletion of 1p36 using the p58 cosmid probe in all 111 cells examined. To exclude the presence of minimal residual disease as an explanation of the constitutional abnormalities, a MYCN locus-specific probe was applied to an aliquot of the same fixed cell suspension. No abnormalities of MYCN were found in any of 60 cells examined, excluding the possibility of tumour cell contamination.

No evidence of chromosomal abnormality, both by karyotype and FISH analysis, was found in the metaphase cells examined from twin 2. Skin biopsy samples were not available from the twins for further constitutional analysis. Constitutional karyotyping of both parents was normal.

**Table I** Constitutional cytogenetic and FISH data for Twin 1

| GTG-band analysis | Number/% of metaphase cells abnormal |
|---|---|
| 46,XX, inv(3)(p23p26) | 2(3.8%) |
| 46,XX, der(1)del(1)(p36 p13.1)dup(1)(q21q44) invinsdup(1)(p13.1q21), inv(3)(p23p26) | 1 (1.9%) |
| 46,XX | 49 (94.3%) |
| **FISH analysis** | **Number/% of metaphase cells abnormal** |
| 46,XX.ish t(1;?17)(p13.1;?q25)(wcp1) | 1 (2.3%) |
| 46,XX.ish del(1)(p13.1p36)(wcp1) | 1 (2.3%) |
| 46,XX.ish (wcp1) | 41 (95.4%) |
| 46,XX.ish del(1)(q44q44)(D1S69-) | 24 (21.6%) |
| 46,XX.ish (D1S69) | 87 (78.4%) |

### DISCUSSION

We have described a pair of monozygotic twins concordant for neuroblastoma. There was no family history of cancer and there were 3 healthy older siblings. Pathological and molecular analysis of the tumours found them to be indistinguishable.

Three hypotheses exist for the concordance of neuroblastoma in this case. Firstly, it could represent separate de novo genetic events occurring independently of each other. The rarity of neuroblastoma and the genetic, clinical and morphological similarity between the tumours makes this possibility extremely unlikely. Secondly, a shared genetic predisposition may have initiated the process of tumorigenesis culminating in 2 independently derived tumours. If this were true, a new germline mutation would be the most likely source given the absence of a family history and the 3 healthy older siblings. Constitutional abnormalities have been found, but only in twin 1, and only in a proportion of haematopoietic cells. Other cell types were not available for confirmation of the cytogenetic findings.

The low level of abnormal cells found in twin 1, suggests either a low-level constitutional mosaicism for abnormalities predisposing her to develop neuroblastoma, or a post-treatment exposure of underlying chromosome instability that had primed her to develop the disease. However, 3 of the 4 rearranged regions discovered in twin 1 (1p, 17q and 3p) involve regions known to be of importance in neuroblastoma tumorigenesis, making random secondary changes extremely unlikely. In total, 3 out of 95 (3.1%) of cells showed abnormalities of chromosome 1p13.1, the region of the N-Ras1 oncogene implicated in neuroblastoma tumorigenesis. This region has previously been reported as a fragile site in lymphocytes studied from patients with neuroblastoma (Rudolph et al, 1988; Vernole et al, 1989). Moreover, a survey of 126 neuroblastoma karyotypes revealed breakpoints at 1p13–21 to be the most common finding (Mertens et al, 1997). The fragile site fra(1)(p13.1) may contribute to neuroblastoma tumorigenesis through secondary effects on 1p36, a region undergoing allele loss in a high proportion of neuroblastomas including those in these 2 children (Brodeur et al, 1981; Gilbert et al, 1982; Schleiermacher et al, 1994; Van Roy et al, 1997). This was the case with both the abnormalities involving 1p detected in twin 1's constitutional samples where both chromosome abnormalities involved the loss of 1p distal to 1p13.1. A further constitutional change involving t(1:17)(p34–36:q11. 2–21) has been described in at least 4

© 2001 Cancer Research Campaign neuroblastoma patients (Laureys et al, 1990; Laureys et al, 1995b; Mitchell et al, 1996; Van Roy et al, 1997). The regions involved in the abnormal cells from twin 1, at 1p13.1 and 17q25, are novel translocation breakpoints and suggest further regions of instability on both chromosomes 1 and 17, possibly contributing to the key stages in neuroblastoma development of loss of 1p36 and gain of distal 17q relative to proximal 17q. Abnormalities of chromosome 3p in neuroblastomas have predominantly been reported as deletions (Breen et al, 2000; Ejeskar et al, 1998; Hallstensson et al, 1997). The inv(3)(p23p26) seen in the metaphase cells from twin 1 is located in the consensus region of deletion in neuroblastoma tumours 3p25.3-3p14.3 (Ejeskar et al, 1998), and therefore might be of some significance. Region 3p is frequently involved in deletions and/or rearrangements in neuroblastoma tumours (Hallstensson et al, 1997). Overall del(3p) is the second most frequently deleted region in this tumour type following del(1p36). A novel region of deletion in the subtelomeric region of chromosome 1 at q44, found in a high proportion (21.6%) of metaphase cells, suggests that this may be a further region of constitutional instability previously unreported in neuroblastoma.

It seems unlikely that twin 2 actually shared the constitutional abnormalities of twin 1, but we were unable to detect them due to sampling error. More than 80 metaphases from twin 2 were analyzed by both G banding and FISH studies using chromosome 1 and 3 probes, and no abnormalities were seen. Therefore, we favour the hypothesis that the chromosomal rearrangements observed represent mosaicism in twin 1 or a genomic instability predisposing to neuroblastoma and acquired in utero following blastocyst division.

A constitutional abnormality present in twin 1 but absent in twin 2 therefore supports a third hypothesis, namely that there was transplacental metastasis of tumour from twin 1 to twin 2. Of note is the finding that twin 2 developed neuroblastoma 6 months following her sibling and the tumour was detected at an early stage, prior to any symptoms developing. This timing would be consistent with micrometastatic deposits of tumour having implanted in twin 2 by the time of delivery. If true, the occurrence of disease in twin 2 in adrenal gland and liver indicates metastasis to adrenal gland, a phenomenon that is not generally recognized in neuroblastoma but is one possible explanation for the rarely described phenomenon of bilateral neuroblastoma.

The early pre-symptomatic detection of tumour in twin 2 illustrates two points. Firstly, although both twins have attained complete remission and although there is no evidence that a large biologically unfavorable tumour is less likely to shrink than a smaller version of the same tumour, twin 2 was not in danger of the severe morbidity associated with the rapidly expanding abdominal mass that her sister had. Waiting until the disease presented itself through the development of a symptomatic mass could have resulted in markedly increased morbidity or mortality related to tumour size. Secondly, the relatively long interval between the development of the 2 tumours indicates that a prolonged period of surveillance may be needed to identify tumours at an early stage.

### ACKNOWLEDGEMENTS

This work was undertaken in part by Great Ormond Street Hospital for Children NHS Trust, which received a proportion of its funding from the NHS Executive. The views expressed in this paper are those of the authors and not necessarily those of the NHS Executive. The Unit of Molecular Haematology is supported by a grant from the Leukaemia Research Fund.

### REFERENCES

Biegel JA, White PS, Marshall HN, Fujimori M, Zackai EH, Scher CD, Brodeur GM and Emanuel BS (1993) Constitutional 1p36 deletion in a child with neuroblastoma. *Am J Hum Genet* **52**: 176–182

Boyd TK and Schofield DE (1995) Monozygotic twins concordant for congenital neuroblastoma: case report and review of the literature. *Pediatr Pathol Lab Med* **15**: 931–940

Breen CJ, O'Meara A, McDermott M, Mullarkey M and Stallings RL (2000) Coordinate deletion of chromosome 3p and 11q in neuroblastoma detected by comparative genomic hybridization. *Cancer Genet Cytogenet* **120**: 44 – 49

Brodeur GM, Green AA, Hayes FA, Williams KJ, Williams DL and Tsiatis AA (1981) Cytogenetic features of human neuroblastomas and cell lines. *Cancer Res* **41**: 4678–4686

Buckley JD, Buckley CM, Breslow NE, Draper GJ, Roberson PK and Mack TM (1996) Concordance for childhood cancer in twins. *Med Pediatr Oncol* **26**: 223–229

Ejeskar K, Aburatani H, Abrahamsson J, Kogner P and Martinsson T (1998) Loss of heterozygosity of 3p markers in neuroblastoma tumours implicate a tumour-suppressor locus distal to the FHIT gene. *Br J Cancer* **77**: 1787–1791

Gilbert F, Balaban G, Moorhead P, Bianchi D and Schlesinger H (1982) Abnormalities of chromosome 1p in human neuroblastoma tumors and cell lines. *Cancer Genet Cytogenet* **7**: 33–42

Hallstensson K, Thulin S, Aburatani H, Hippo Y and Martinsson T (1997) Representational difference analysis and loss of heterozygosity studies detect 3p deletions in neuroblastoma. *Eur J Cancer* **33**: 1966–1970

Kushner BH and Helson L (1985) Monozygotic siblings discordant for neuroblastoma: etiologic implications. *J Pediatr* **107**: 405–409

Laureys G, Speleman F, Opdenakker G, Benoit Y and Leroy J (1990) Constitutional translocation t(1;17)(p36;q12–21) in a patient with neuroblastoma. *Genes Chromosomes Cancer* **2**: 252–254

Laureys G, Speleman F, Versteeg R, van der Drift P, Chan A, Leroy J, Francke U, Opdenakker G and Van Roy N (1995a) Constitutional translocation t(1;17)(p36.31–p36.13;q11.2–q12.1) in a neuroblastoma patient. Establishment of somatic cell hybrids and identification of PND/A12M2 on chromosome 1 and NF1/SCYA7 on chromosome 17 as breakpoint flanking single copy markers. *Oncogene* **10**: 1087–1093

Laureys G, Versteeg R, Speleman F, van der Drift P, Francke U, Opdenakker G and Van Roy N (1995b) Characterisation of the chromosome breakpoints in a patient with a constitutional translocation t(1;17)(p36.31–p36.13;q11.2–q12) and neuroblastoma. *Eur J Cancer* **4**: 523–526

Mancini AF, Rosito P, Faldella G, Serra L, Vallicelli R, Vecchi V, Vivarelli F and Paolucci G (1982) Neuroblastoma in a pair of identical twins. *Med Pediatr Oncol* **10**: 45–51

Mead RS and Cowell JK (1995) Molecular characterization of a (1;10)(p22;q21) constitutional translocation from a patient with neuroblastoma. *Cancer Genet Cytogenet* **81**: 151–154

Mertens F, Johansson B, Hoglund M and Mitelman F (1997) Chromosomal imbalance maps of malignant solid tumors: a cytogenetic survey of 3185 neoplasms. *Cancer Res* **57**: 2765–2780

Mitchell EL, McNally K and Kelsey A (1996) Involvement of chromosomes 1 and 17 in a case of neuroblastoma. *Pediatr Hematol Oncol* **13**: 457–461

Rudolph B, Harbott J and Lampert F (1988) Fragile sites and neuroblastoma: fragile site at 1p13.1 and other points on lymphocyte chromosomes from patients and family members. *Cancer Genet Cytogenet* **31**: 83–94

Schleiermacher G, Peter M, Michon J, Hugot JP, Vielh P, Zucker JM, Magdelenat H, Thomas G and Delattre O (1994) Two distinct deleted regions on the short arm of chromosome 1 in neuroblastoma. *Genes Chromosomes Cancer* **10**: 275–281

Van Roy N, Laureys G, Van Gele M, Opdenakker G, Miura R, van der Drift P, Chan A, Versteeg R and Speleman F (1997) Analysis of 1;17 translocation breakpoints in neuroblastoma: implications for mapping of neuroblastoma genes. *Eur J Cancer* **33**: 1974–1978

Vernole P, Tedeschi B, Caporossi D and Nicoletti B (1989) Fragile site 1p13.1 in neuroblastoma patients. *Cancer Genet Cytogenet* **40**: 135–136

© 2001 Cancer Research Campaign 
