# Neuroblastoma in monozygotic twins – a case of probable twin to twin metastasis

**DOI:** 10.1038/sj.bjc.6600308

**Published:** 2002-05-03

**Authors:** J Anderson, H Kempski, L Hill, D Rampling, T Gordon, A Michalski

## Abstract

*British Journal of Cancer* (2002) **86**, 1666–1666. DOI: 10.1038/sj/bjc/6600308 www.bjcancer.com

© 2002 Cancer Research UK

## 

**Correction to:** *British Journal of Cancer* (2001) **85**, 493-496. DOI: 10.1054/bjoc.2001.1979

The authors would like to acknowledge the Research into Childhood Cancer charity (Gibraltar) (RICC) for funding for the above project.

